# The six-year decomposition of coarse woody debris drives shifts in soil fungal communities in subtropical forests

**DOI:** 10.3389/fmicb.2025.1544163

**Published:** 2025-09-22

**Authors:** Nan Wang, Binle Ding, Ruyi Zhang, Hui Chen, Tingsi Xie, Shangbin Bai, Hua Chen, Xiaocheng Pan

**Affiliations:** ^1^Jiyang College, Zhejiang A&F University, Zhuji, China; ^2^Forestry and Biotechnology College, Zhejiang A&F University, Linan, China; ^3^Department of Biology, University of Illinois Springfield, Springfield, IL, United States

**Keywords:** coarse wood debris, fungal community, trophic modes, tree species, carbon sequestration

## Abstract

**Introduction:**

Coarse woody debris (CWD) plays a vital role in forest ecosystems, serving as a reservoir for carbon sequestration. While global climate change is expected to exacerbate forest disturbances and lead to a significant accumulation of CWD, the effect of CWD decomposition on the composition, diversity and functional traits of soil fungal communities remains unclear, especially for subtropical forests with high tree species diversity.

**Methods:**

Here, we conducted a 6-year *in situ* field experiment (2018–2024) in a subtropical evergreen broad-leaved forest in southern China. We used high-throughput sequencing and qPCR to examine how decomposition of three dominant tree species (conifer, broadleaved, and woody monocot moso bamboo) influences soil fungal composition, and applied the FUNGuild tool to infer fungal trophic modes and functional groups from sequencing data. We found that 6 years of CWD decomposition significantly increased soil organic carbon (SOC), dissolved organic carbon (DOC), and microbial biomass carbon (MBC) while reducing soil pH. Bamboo CWD showed the highest SOC and MBC accumulation.

**Results:**

High-throughput sequencing of the ITS1 region indicated a statistically significant increase in α-diversity and a marked differentiation in β-diversity of fungal communities following decomposition. Taxonomic analysis identified Ascomycota and Basidiomycota as the dominant fungal phyla. CWD decomposition was associated with observable differences in taxonomic composition, specifically an increase in the Basidiomycota-to-Ascomycota ratio. Key gener as such as Geminibasidium, *Trichoderma*, and *Trechispora* exhibited species-specific responses to both CWD decomposition and tree species identity. Functional analysis via FUNGuild revealed an increased relative abundance of taxa predicted to be saprotrophic, alongside a decreased relative abundance of taxa inferred to be symbiotrophic. Soil pH and SOC emerged as the primary factors influencing fungal community structure.

**Discussion:**

These findings highlight the critical role of CWD in shaping soil fungal communities and their inferred functional traits, underscore the influence of tree species identity on fungal assembly, and provide insights into stable carbon sequestration stability in subtropical forests.

## 1 Introduction

Coarse woody debris (CWD) is defined as dead woody material ≥1 m in length and >10 cm in diameter at the thinner end (Harmon et al., 2020), and it is a key component of forest ecosystems. Globally, it accounts for 5%–18% of the forest carbon pool (Marzano et al., 2013; Chen et al., 2021) and supports critical ecological, including harboring biodiversity, promoting tree regeneration (Kumar et al., 2020; Müller et al., 2020), and facilitating forest carbon sequestration with a particular role in soil carbon input (Pan et al., 2011; Russell et al., 2015; Shorohova et al., 2022). Global climate change is expected to intensify forest disturbances (e.g., hurricanes, wildfires, droughts, insect outbreaks) and interspecies competition, which will significantly increase tree mortality (Allen et al., 2015; Bradford et al., 2024). This elevated mortality drives the transfer of woody carbon from live to dead pools, a process projected to accelerate in the future and potentially alter forest ecosystem carbon dynamics (Silva et al., 2016). Therefore, understanding the decomposition of aboveground CWD and the fate of CWD-derived compounds in forest soil has become a critical focus of forest ecology research under global climate change.

The complete decomposition of CWD can take up to hundreds of years, and the key factors influencing decomposition may change dynamically over the decomposition period. Currently, research on CWD decomposition rates, nutrient dynamics, and the community assembly processes of associated microorganisms is mainly conducted through methods such as short-term observation, long-term monitoring, chronosequence, and laboratory culture. Short-term observation, which usually lasts 1–5 years, has low time cost but only presents short-term decomposition process (Seibold et al., 2021). Long-term monitoring is one of the commonly used methods in current research. It is appropriate for monitoring changes in environment during decomposition and facilitates the disentanglement of decomposition mechanisms. However, due to the high time cost, only a small number of research teams have been able to implement long-term monitoring (Seibold et al., 2023). For instance, Germany’s BE Long Dead project, launched in 2009 (Kahl et al., 2017), and the Netherlands’ LOGLIFE project, established in 2012 (Cornelissen et al., 2012), have systematically explored the key factors influencing the decomposition of CWD during the early and middle decomposition stages, as well as the associated biodiversity, through standardized experimental designs and long-term *in situ* studies. These projects have provided valuable findings for global research on woody debris decomposition in forest ecosystems. Chronosequence, which substitutes space for time, enables an understanding of the whole decomposition process through a single survey. However, while it can only access the trend of decomposition, it is characterized by low accuracy (Harmon et al., 1986). Laboratory culture, the decomposition environment, decomposer community, and substrate quality can be strictly controlled. However, many microorganisms can not be cultured in the lab, and the decomposition process can be different with that in the field (Valášková et al., 2009).

Coarse woody debris serves as a critical intermediary in forest ecosystems, linking vegetation and soil carbon pools through complex decomposition processes. As it decays, carbon is primarily released as carbon dioxide via microbial respiration (Hollands et al., 2022). Simultaneously, carbon enters soil organic matter through leaching, physical fragmentation, and enzymatic breakdown by decomposers. These processes transfer carbon from decaying wood to the dissolved organic carbon (DOC) pool, which in turn contributes to the formation and accumulation of soil organic carbon (SOC) (Wiebe et al., 2014). Carbon conversion during CWD decomposition is shaped by multiple factors, including wood traits, environmental conditions, and the microbial communities driving decomposition (Kahl et al., 2017). For instance, tree species differ in decomposition rates, which affects the quantity and form of carbon released into soil. Moreover, CWD decomposition can boost soil microbial activity, enhancing the transformation of carbon into stable SOC that persist in soil over long periods (Xie et al., 2022).

Soil microorganisms are key drivers of the terrestrial carbon cycle, with dual roles. One is mediating the transfer of aboveground biomass carbon to subsurface environments and promoting soil organic carbon decomposition and cycling via enzymatic and metabolic processes; the other is contributing assimilated carbon to soil pools through growth, reproduction, and death, which deposits it as stabilized microbially-derived organic matter. CWD decomposition and soil microorganisms maintain a complex relationship. CWD provides critical habitat for soil microorganisms across life stages (Larrieu et al., 2014), supporting the structure and function of soil biological communities (Pan et al., 2011), CWD gradually releases essential nutrients, providing a long-term substrate for microbial sustenance and growth (Oishi, 2024). At the same time, CWD alters soil nutrient content, availability, and pH in its vicinity (Zalamea et al., 2007), which in turn modulates microbial community structure and diversity. Thus, comprehensive and quantitative studies are essential to explore how CWD decomposition in subtropical forests impacts soil microbial communities and their functions.

Soil fungi are a vital component of the soil microbiome and perform important functions in forest ecosystems, particularly in decomposing refractory plant litter and mineralizing soil carbon (C) and nitrogen (N) (Phillips et al., 2013; Li et al., 2024). Fungi involved in litter decomposition exhibit diverse functional capabilities. White-rot fungi (a major group of lignin-decomposers) produce extracellular enzymes to degrade cellulose and lignin (Floudas et al., 2012), while soluble sugar-decomposing fungi (e.g., yeasts) rapidly colonize and metabolize simple sugars, enabling efficient nutrient recycling from plant residues (Treseder and Lennon, 2015). Fungal community is shaped by abiotic factors, such as spatial heterogeneity and edaphic conditions (van der Linde et al., 2018), and biotic factors, including host species (Steidinger et al., 2019).

Over the past decades, microbial communities has been increasingly recognized as critical for understanding decomposition processes, driven by recent advances in molecular tools and analytical methods (Bani et al., 2018; Glassman et al., 2018; Maillard et al., 2022). For example, microbial communities mediate both direct and indirect effects of climate on decomposition, which challenges traditional paradigms of focusing solely on abiotic drivers (Bradford et al., 2017). Shifts in microbial substrate preferences and changes in the proportion of microbial communities linked to different lifehistory strategies (r- or K-strategists) strongly affect deadwood structural stability (Malik et al., 2020; Zeng et al., 2022). Generally, r-strategists microbes thrive in environments with rich labile C due to their rapid growth rates, whereas K-strategists microbes are more efficient at using low-availability C sources in stable environments (Chen H. et al., 2022; Chen Y. et al., 2022).

Coarse woody debris acts as a critical host for soil fungi and contributes to ecosystem nutrient cycling. Host tree species also affect fungal community composition and trophic guilds (Bachelot et al., 2017). However, the specific responses of fungal populations with distinct functional traits and life-history strategies to deadwood decomposition remain unclear. Investigating the dynamics of soil fungal communities during CWD decomposition through the r/K-selection theory can provide critical insights into how these populations adapt to the decomposition process. This approach is essential for advancing understanding of the influence of fungal communities on fundamental ecosystem processes such as nutrient cycling, soil formation, and carbon sequestration. These processes are pivotal for maintaining ecosystem stability, functionality, and resilience.

Moso bamboo (*Phyllostachys edulis*) is a tall clonal grass with a tree-like growth habit that forms distinct forest ecosystems, predominantly in subtropical China. It has exceptional carbon sequestration capacity, playing a critical role in carbon cycling and climate change mitigation (Chiti et al., 2024). Its ability to store amounts of atmospheric carbon highlights its ecological importance for regulating the subtropical carbon balance and supporting ecosystem sustainability. Moso bamboo spreads mainly through vigorous underground rhizomes (bamboo shoots), allowing it to invade and gradually replace adjacent coniferous and broadleaf forests (Liu et al., 2023). Meanwhile, ecological succession and renewal processes within moso bamboo forests, along with human disturbances, have led to substantial CWD accumulation in these subtropical forest ecosystems. However, little research has focused on fallen wood (from the dieback and mortality of trees displaced by moso bamboo expansion) affects soil microbial communities.

In this study, we conducted a randomized controlled experiment in a subtropical forest in Eastern China. We compared four treatments, including soil without CWD and soils under decomposing logs of three different tree species to investigate the successional dynamics of fungal abundance, diversity and community composition during CWD decomposition and their coupling with edaphic physicochemical properties. We also examined how the content of DOC, SOC and microbial biomass beneath CWD varied among tree species including conifers (*Pinus massoniana* Lamb.), broadleaf specie (*Schima superba Gardner & Champ.*) and bamboo (*Phyllostachys edulis*) after 6 years of decomposition. Our hypotheses that (1) decomposition of bamboo deadwood increases microbial biomass carbon (MBC) accumulation, enhancing SOC stability and sequestration; (2) fungal diversity, life strategies and trophic modes at the soil-log interface increase with deadwood decomposition; and (3) taxonomic composition and trophic modes of fungal communities in soil beneath CWD differ significantly among tree species.

## 2 Materials and methods

### 2.1 Study sites

The experiment was established in early 2018 at Wuxie National Forest Park, Zhejiang Province, China (29.72°N, 120.05°E) with an average elevation of 220 m. The study area lies in the mid-subtropical monsoon climate zone, with an average annual temperature of 17.6°C. The lowest temperature occurs in January at approximately 1.2°C and the highest in August at around 36.2°C. Average annual precipitation from 2018 to 2023 is about 1,298 mm, mainly concentrated from May to August. The soil is dominated by mountainous red and yellow loams. The main vegetation is subtropical evergreen broad-leaved forest, consisting of *Pinus massoniana* Lamb., *Cunninghamia lanceolata* Lamb. & Hook., *Quercus glauca* Thunb., and *Schima superba* Gardner & Champ., *Phyllostachys edulis* (moso bamboo). *Phyllostachys edulis* has been widely reported to invade the nearby evergreen broadleaf forests in this region, forming transition areas and causing significant tree mortality.

### 2.2 Experimental design and sampling

In 2018, a long-term deadwood decomposition experiment platform was established in a mixed forest within the study area. Three repeated plots (10 m × 10 m each) were set up based on slope, with at least 50 m between adjacent plots. Fresh logs (∼1.5 m in length, *N* = 25–30) of each target tree species were randomly placed in the plots, and the distance between any two logs was at least 1 m (Supplementary Figure 1). All downed logs were laid horizontally on the ground to maximize their surface contact with the soil. Areas without deadwood (untreated) were designated as controls (CK). We defined three soil ground: SS refers to the soil beneath *Schima superba* deadwood, PM to the soil under *Pinus massoniana* deadwood, and PE to the soil covered by *Phyllostachys edulis* deadwood. The wood samples were collected from fresh logs of *Pinus massoniana, Schima superba, and Phyllostachys edulis* to determine their initial chemical composition (Supplementary Table 1). The sites of all forestlands were similar in terms of altitude, slope position, and aspect, and the soils of all sites had a loamy texture (Supplementary Table 2).

Soil samples were collected in June 2024 directly beneath the center and ends of each downed log, at a 0–10 cm depth after removing surface litter. Collected samples were homogenized via quartering and divided into two aliquots. The first aliquot was placed in sterile self-sealing bags, air-dried in the lab, cleared of stones and debris, and sieved (2 mm) for soil property analysis. The second aliquot was immediately stored in 10 ml centrifuge tubes on dry ice, then ground, homogenized, and stored at −80 °C for the high-throughput sequencing of the soil fungal community and functional group analysis (Zhang et al., 2016). For each fallen log type, all soil samples were collected from spatially separated plots. Each plot had 3 biological replicates, and per sample had 2 technical replicates. Samples from decomposing log edges were explicitly excluded to avoid non-independent observations.

### 2.3 Chemical analysis of soil samples

Soil pH was determined in water (soil-to-water ratio 1:2.5, w/v) using a PHS-3C pH meter (Mettler Toledo, Switzerland). SOC was measured by the potassium dichromate–sulfuric acid oxidation method (K_2_Cr_2_O_7_–H_2_SO_4_). DOC was extracted with ultrapure deionized water, filtered through a 0.45 μm membrane, and analyzed using a multi N/C 2100 TOC analyzer (Analytik Jena GmbH, Jena, Germany). Soil microbial biomass carbon and nitrogen (MBC and MBN, mg kg^–1^) were measured with the chloroform fumigation–extraction method. Approximately 4 g of fresh soil was divided into two portion. One portions was fumigated with ethanol-free chloroform for 24 h at 25°C, and the other was kept unfumigated as a control. Both portions were extracted with 0.5 M K_2_SO_4_ by shaking for 1.5 h on an overhead shaker at ambient temperature, followed by filtration using filter paper. Carbon and nitrogen contents in the extracts were analyzed. MBC and MBN were calculated as the differences between fumigated and unfumigated samples divided by the extraction efficiency factors (Brookes et al., 1985).

### 2.4 DNA extraction, quantitative PCR (qPCR), and sequencing

Fungal genomic DNA was extracted from 0.5 g soil using the E.Z.N.A.^®^ Soil DNA Kit (Omega Bio-tek, Norcross, GA, USA) following the manufacturer’s instructions. The quantity and concentration of extracted DNA were determined via 1% (w/v) agarose gel electrophoresis and a NanoDrop spectrophotometer (NanoDrop Technologies, Wilmington, DE, USA). The fungal internal transcribed spacer (ITS) region was amplified with the primer pair ITS1F (5′-CTTGGTCATTTAGAGGAAGTAA-3′) and ITS2R (5′-GCTGCGTTCTTCATCGATGC-3′) using the protocol described by Li et al. (2021) (Li et al., 2021).

PCR was performed using the ABI GeneAmp^®^ 9700 PCR System (Applied Biosystems, USA) with a 20 μL reaction mixture containing 4 μL 5 × FastPfu Buffer, 2 μL 2.5 mM dNTPs, 0.4 μL FastPfu Polymerase, 0.8 μL each of 5 μM the forward and reverse primers, 0.2 μL bovine serum albumin, 1 μL template DNA and 10.8 μL double-distilled H_2_O. ITS PCR cycling conditions included denaturation at 95 °C for 3 min, 35 cycles of 95 °C for 30 s, 55 °C for 30 s and 72 °C for 45 s, and a final elongation at 72 °C for 10 min. DNA from each soil sample was amplified three times by PCR. Amplification products were separated by electrophoresis on 2% agarose gels and purified using an AxyPrep DNA Gel Extraction Kit (Axygen Biosciences, USA). PCR products were quantified with QuantiFluor™-ST (Promega, USA). Final libraries were sequenced on the Illumina MiSeq PE300 platform (Majorbio Biotechnology Co., Ltd. Company, Shanghai, China).

### 2.5 Bioinformatics

Raw sequencing data were spliced, quality-controlled using FASTP and FLASH, and filtered using QIIME2 (Caporaso et al., 2010). Denoising, error estimation, chimera removal and merging were performed using DADA2. Amplicon sequence variants (ASVs) constructed by DADA2 were then clustered into operational taxonomic units at a 97% similarity cut off by UPARSE (Rognes et al., 2016). Taxonomic classification was conducted with mothur (Schloss et al., 2009) using its Naive Bayesian classifier, with the UNITE database (version 9.0) and SILVA database (Quast et al., 2012) as references for fungal ITS sequences. All sequencing data were deposited in the NCBI SRA database under accession number PRJNA1186394 (SAMN44755544-44755555) Functional guilds of the fungal community were tentatively assigned with high, moderate and low confidence using the FUNGuild algorithm (Nguyen et al., 2016).

### 2.6 Statistical analyses

One-way analysis of variance (ANOVA) with Duncan’s test (*P* < 0.05) was used in SPSS 23.0 to assess differences in soil properties, fungal diversity and abundance among soil grounds of different tree species. The number of ASVs across samples was analyzed in R (Version 3.3.1) using the “Venn” package, with a Venn diagram to visually compare compositional similarity and overlap of fungal ASVs. The α diversity indices (Chao1, Shannon-Wiener) and species accumulation curves were calculated in Mothur and visualized in R (version 3.3.1) using the “ggplot2” package. β diversity was analyzed in R (version 3.3.1) through Principal Coordinate Analysis (PCoA) ordinations based on Bray–Curtis distance. Non-parametric permutational multivariate analysis of variance (Adonis) was utilized to investigate significant variations. Taxonomic composition at phylum and genus levels was analyzed in QIIME2 using the classify-sklearn algorithm and Naive Bayes classifier, with fungi identified against the UNITE database (Release 8.0)^1^ (Abarenkov et al., 2010). Redundancy analysis (RDA) was performed in Canoco5.0 (Microcomputer Power, USA) to examine correlations between soil parameters and fungal communities, with statistical significance evaluated through Monte Carlo permutation tests (499 permutations; *P* < 0.05). The Mantel test in the ggcor package of R (version 3.3.1) explored correlations between genus-level fungal communities and soil parameters. Fungal trophic modes and functional groups were determined using FUNGuild software with uploaded the fungal ASV data.

## 3 Results

### 3.1 Soil carbon, pH and microbial biomass

After 6 years of decomposition, soils beneath deadwood had significantly higher DOC and SOC concentrations than soils without deadwood (control, CK) (*P* < 0.05), while soil pH showed the opposite trend and decreased in the presence of deadwood (Table 1). Tree species had significant effects on soil pH, DOC, and SOC (Supplementary Table 3). Specifically, SS and PE reduced soil pH by 0.42 and 0.30 units respectively, compared to CK (*P* < 0.05), whereas PM deadwood caused a non-significant downward trend in soil pH. DOC concentrations was highest beneath PM deadwood, significantly higher than that beneath SS and PE deadwood (*P* < 0.05) and 50.18% higher than CK. In contrast, SOC was significantly higher in soils beneath PE deadwood, increasing by 135.57% compared to CK. CWD decomposition significantly increased soil microbial biomass. MBC was highest under PE deadwood, which was 54.53, 9.81, and 83.56% higher than CK (Table 1 and Supplementary Table 3).

**TABLE 1:** Effects of different treatments on soil physicochemical properties and microbial biomass.

Treatment	pH	DOC (mg⋅kg^–1^)	SOC (g⋅kg^–1^)	MBN (mg⋅kg^–1^)	MBC (mg⋅kg^–1^)
CK	5.85 ± 0.12a	45.03 ± 2.95c	28.76 ± 10.24b	24.90 ± 0.51c	265.16 ± 24.24c
SS	5.43 ± 0.04b	54.70 ± 4.26b	38.57 ± 6.19b	47.55 ± 2.78a	409.76 ± 13.99b
PM	5.79 ± 0.16a	67.63 ± 1.19a	43.26 ± 6.76b	39.66 ± 1.81b	291.17 ± 15.86c
PE	5.55 ± 0.02b	55.60 ± 1.55b	67.75 ± 16.67a	42.44 ± 3.35ab	486.73 ± 18.50a

Different lowercase letters indicate significant differences *P* < 0.05 among tree species. Data are presented as mean ± standard deviation (*n* = 3). SOC, soil organic carbon; DOC, dissolved organic carbon; MBC, microbial biomass carbon; MBN, microbial biomass nitrogen; Ck, Control (soil group without deadwood); SS, *Schima superba* (soil group beneath *Schima superba* deadwood); PM, *Pinus massoniana* (soil group under *Pinus massoniana* deadwood); PE, *Phyllostachys edulis* (soil group beneath *Phyllostachys edulis* deadwood).

### 3.2 Soil fungal community diversity and composition

**FIGURE 1:**
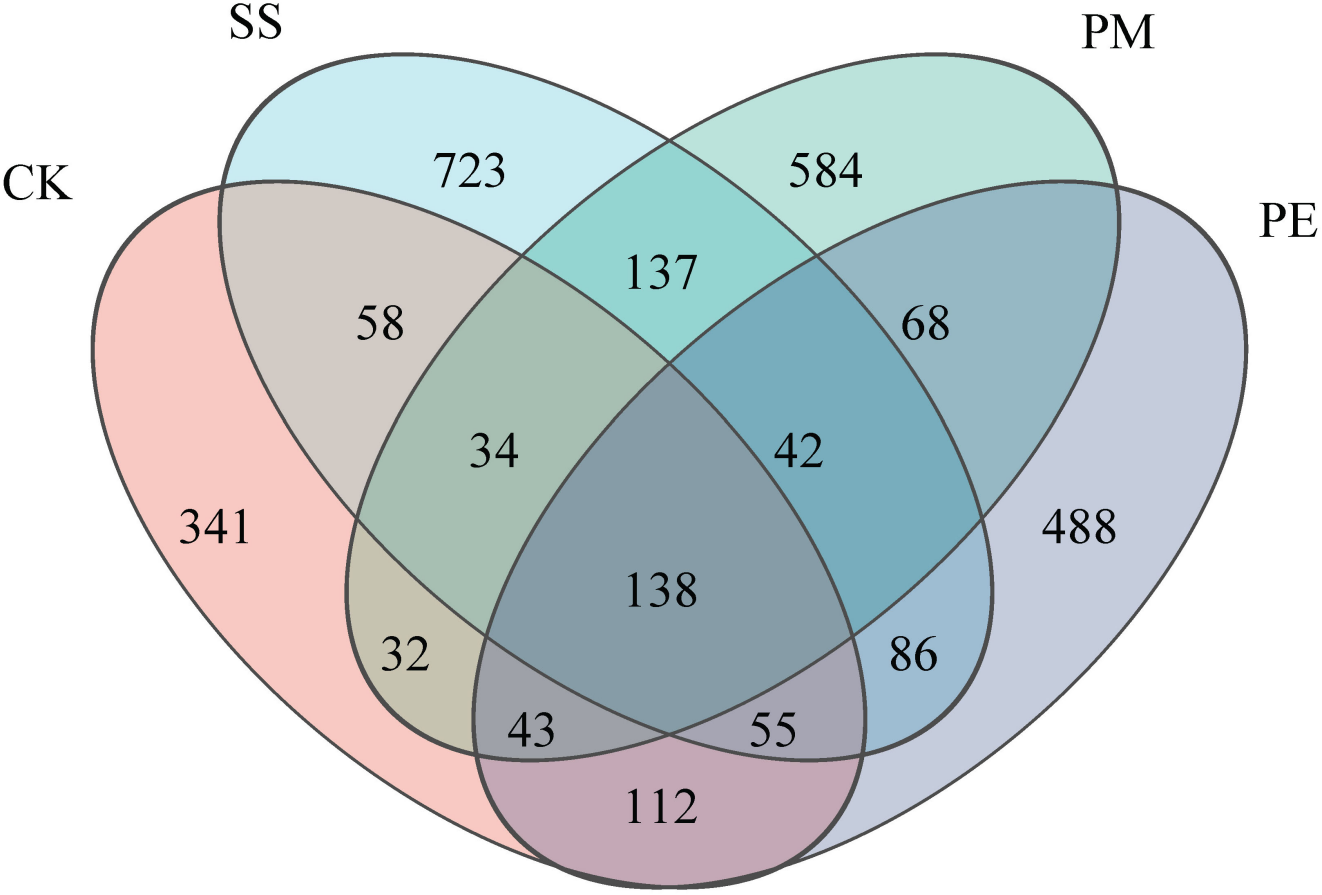
Venn to show the cluster distribution of the fungi in the decomposition of different fallen logs based on ASV level. Ck, Control (soil group without deadwood); SS, *Schima superba* (soil group beneath *Schima superba* deadwood); PM, *Pinus massoniana* (soil group under *Pinus massoniana* deadwood); PE, *Phyllostachys edulis* (soil group beneath *Phyllostachys edulis* deadwood).

**FIGURE 2:**
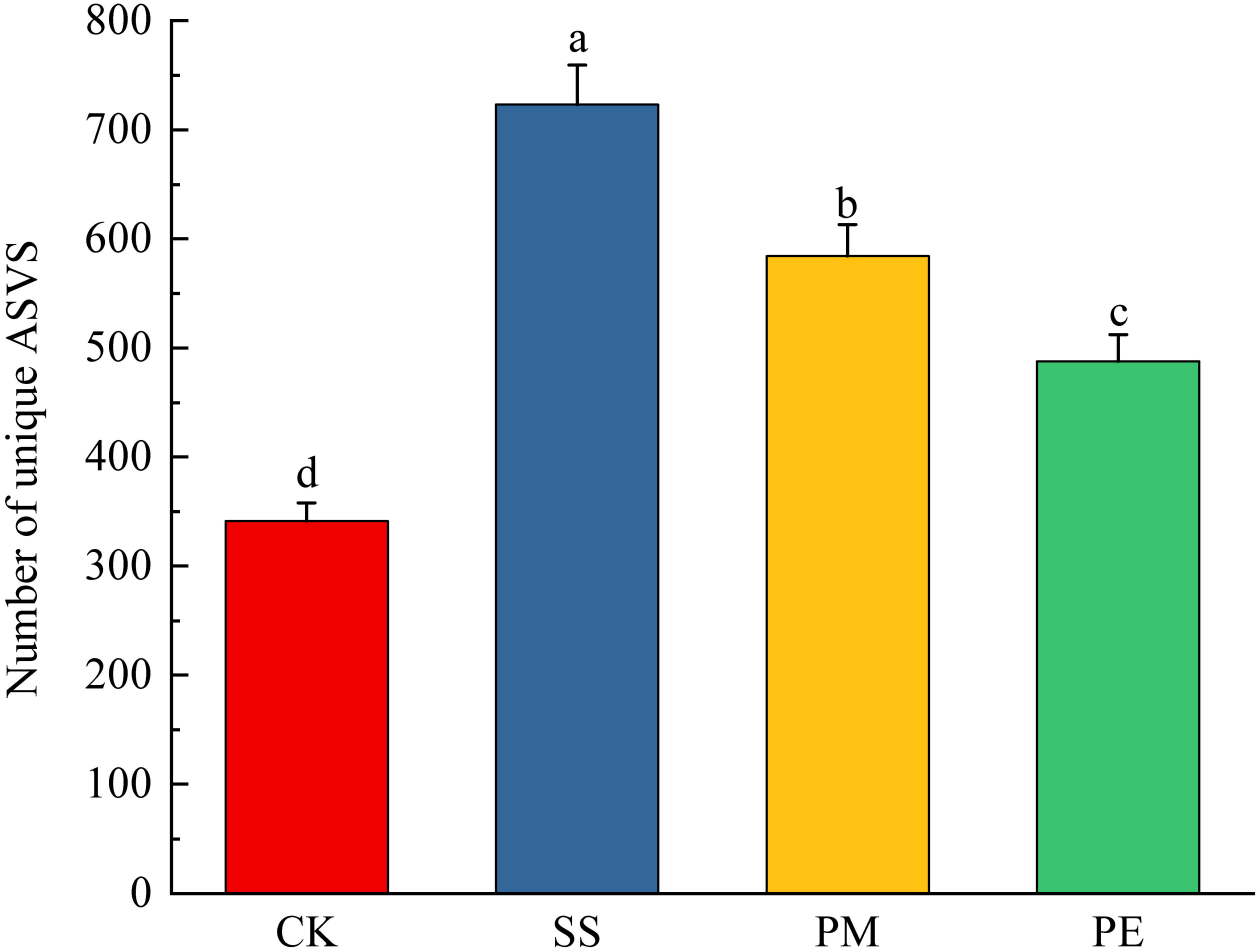
Number of unique amplicon sequence variants (ASVs) in soils under different fallen log types. Ck, Control (soil group without deadwood); SS, *Schima superba* (soil group beneath *Schima superba* deadwood); PM, *Pinus massoniana* (soil group under *Pinus massoniana* deadwood); PE, *Phyllostachys edulis* (soil group beneath *Phyllostachys edulis* deadwood).

**FIGURE 3:**
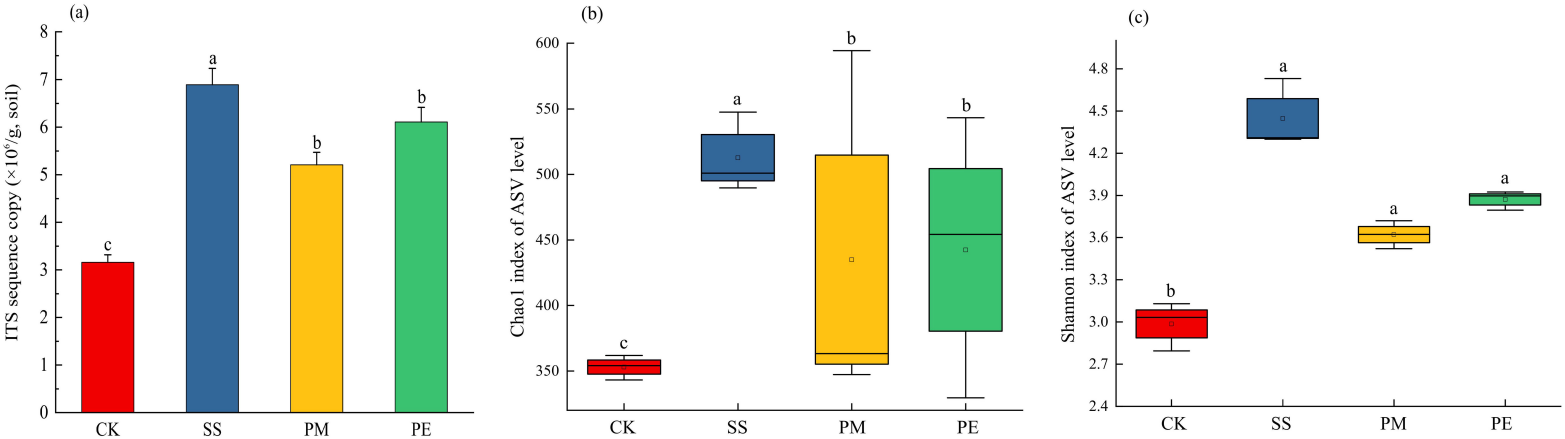
Fungal abundance and Alpha diversity indices under different fallen log types. Fungal abundance indicated by ITS gene copy number **(a),** Chao1 index **(b)** and Shannon index **(c)** Ck, Control (soil group without deadwood); SS, *Schima superba* (soil group beneath *Schima superba* deadwood); PM, *Pinus massoniana* (soil group under *Pinus massoniana* deadwood); PE, *Phyllostachys edulis* (soil group beneath *Phyllostachys edulis* deadwood).

A total of 570,457 reads and 5,224 amplicon sequence variants (ASVs) were obtained from all samples (Supplementary Table 4). After filtering and normalization, these were reduced to 448,057 reads and 2,941 ASVs. Plateaued rarefaction curves demonstrated that most fungal community diversity had been captured (Supplementary Figure 2). After decomposition, the number of unique ASVs increased significantly (*P* < 0.05; Figure 2). The proportions of unique ASVs beneath SS, PM, and PE deadwood rose to 24.5, 19.8, and 16.6% respectively (*P* < 0.05, Figures 1, 2). Fungal ITS genes abundance in soils under different treatments was quantified by qPCR (Figure 3a). Gene copy numbers ranged from 3.16 × 10^6^ to 6.89 × 10^6^ copies g^–1^ soil. CK had the lowest abundance (3.16 × 10^6^ copies g^–1^), 3.73 × 10^6^ copies g^–1^ lower than the highest value (recorded for SS, 6.89 × 10^6^ copies g^–1^). SS differed significantly from all other treatments, whereas PM and PE did not differ from each other but were both significantly higher than CK (*P* < 0.05). CWD decomposition increased the Chao1 richness and Shannon diversity indices of the soil fungal community (Figure 3b, c). These indices also varied significantly among tree species, with soils beneath SS deadwood showing higher Chao1 richness and Shannon diversity than those beneath PM and PE deadwood (Figure 3b, c).

**FIGURE 4:**
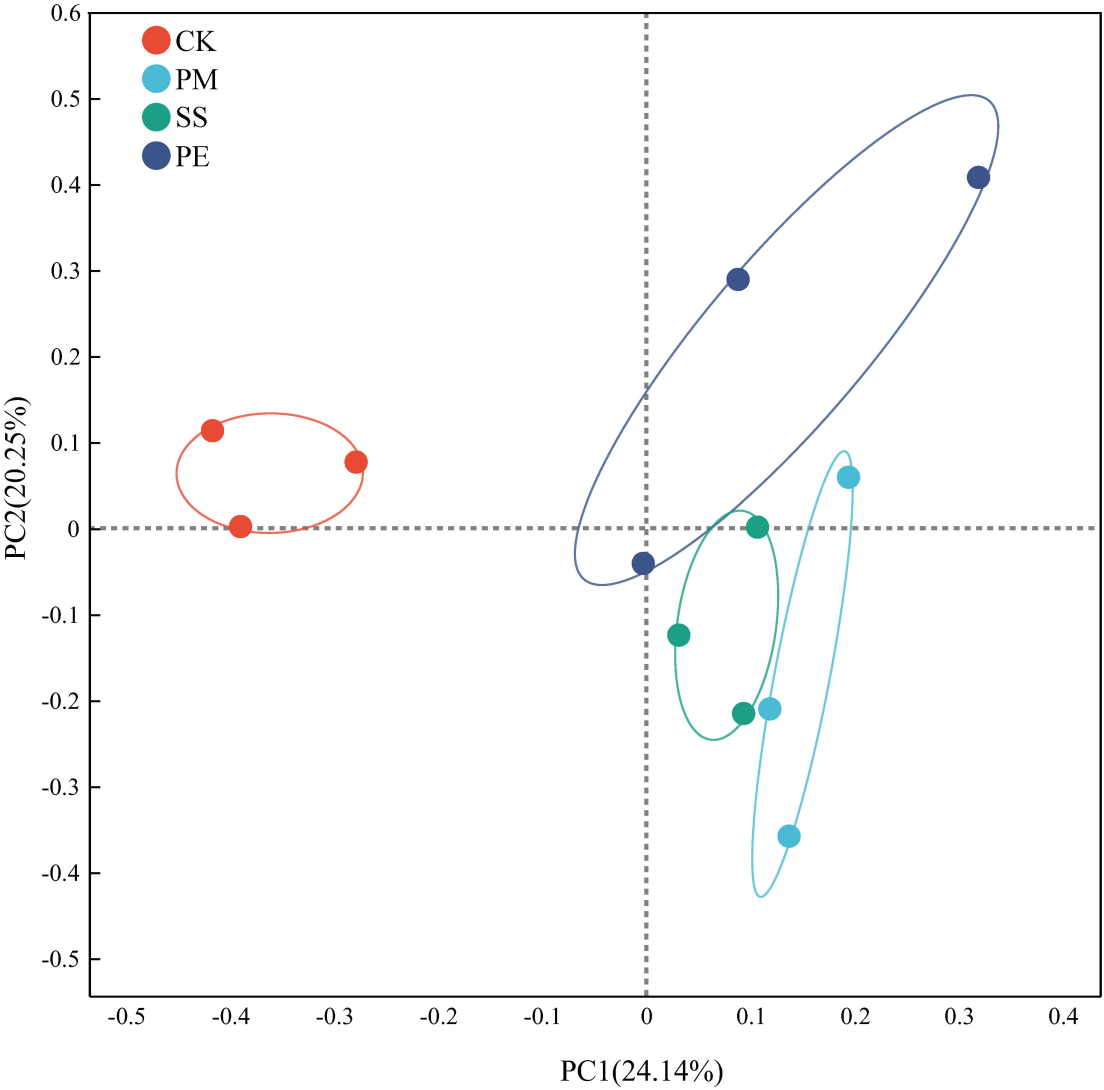
Principal coordinates analysis (PCoA) of soil fungal communities under different fallen log types, based on the Bray-Curtis distance matrix. Ck, Control (soil group without deadwood); SS, *Schima superba* (soil group beneath *Schima superba* deadwood); PM, *Pinus massoniana* (soil group under *Pinus massoniana* deadwood); PE, *Phyllostachys edulis* (soil group beneath *Phyllostachys edulis* deadwood).

β-Diversity analysis using principal co-ordinates analysis (Figure 4) at the ASV level showed that axes 1 and 2 explained 24.1% and 20.3% of the overall variances among the plots, respectively. A clear separation was observed between CWD plots (SS, PM and PE) and CK plots (Figure 4). PERMANOVA indicated substantial changes in fungal community structure dissimilarity after 6 years of decomposition (*r* = 0.429, *P* = 0.004).

**FIGURE 5:**
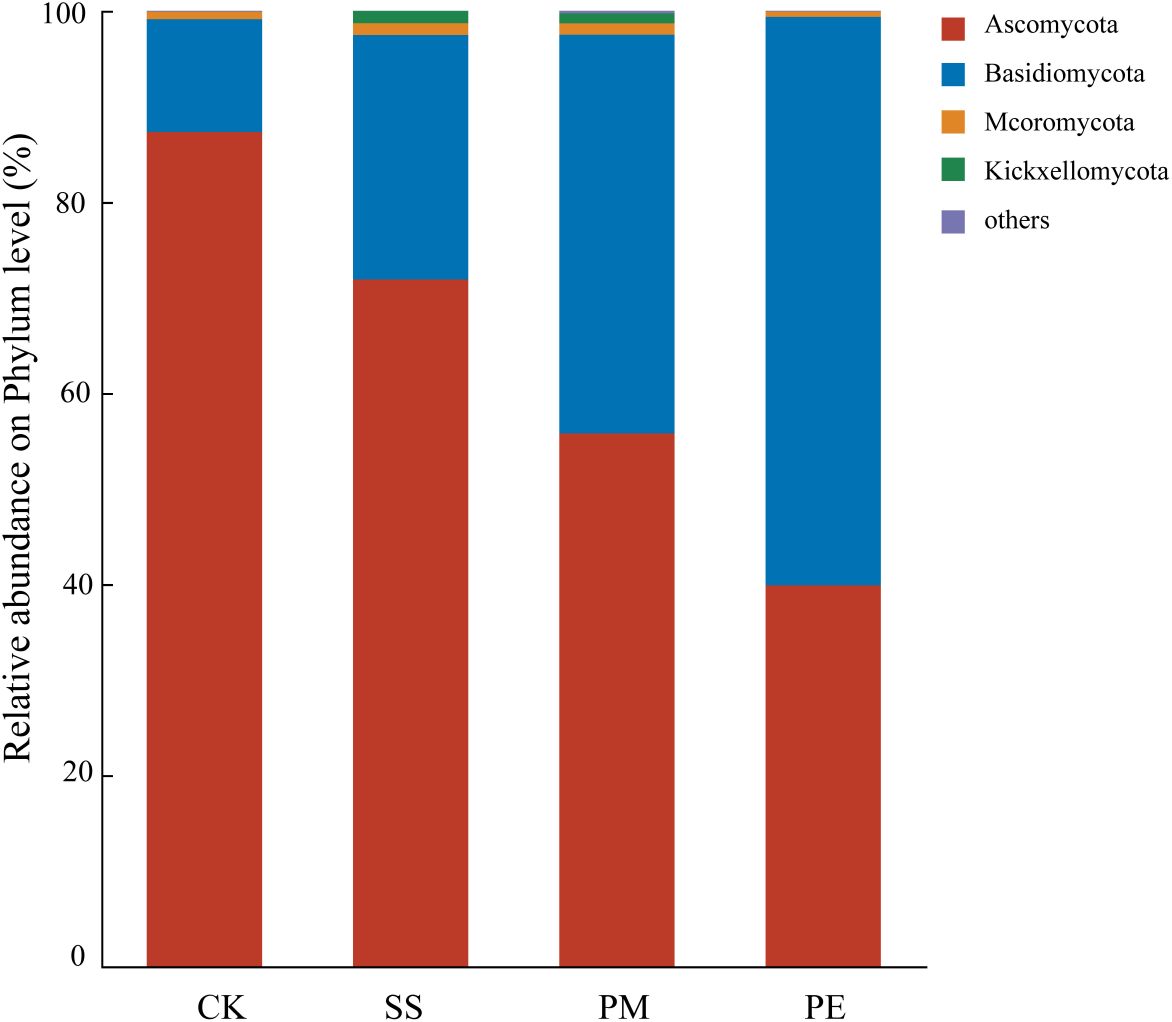
Relative abundance of soil fungi at the phylum level under different fallen log types. Ck, Control (soil group without deadwood); SS, *Schima superba* (soil group beneath *Schima superba* deadwood); PM, *Pinus massoniana* (soil group under *Pinus massoniana* deadwood); PE, *Phyllostachys edulis* (soil group beneath *Phyllostachys edulis* deadwood).

In total of 14 fungal phyla and 599 genera were identified, among which 4 phyla and 10 genera had relative abundances exceeding 1%. Ascomycota and Basidiomycota were the dominant phyla in soil samples, with mean relative abundances of 63.71 and 34.67%, respectively, and together accounted for 97.49 to 99.38% of all fungal sequences (Figure 5). CWD decomposition significantly decreased the relative abundance of Ascomycota while increasing that of Basidiomycota (Figure 5). Specifically, the relative abundance of Ascomycota in soils beneath SS, PM, and PE deawood decreased by 17.68, 35.89, and 54.34%, respectively. In contrast, the relative abundance of Basidiomycota beneath PE deadwood was higher than that beneath SS and PM deadwood (Figure 5 and Supplementary Table 5).

**FIGURE 6:**
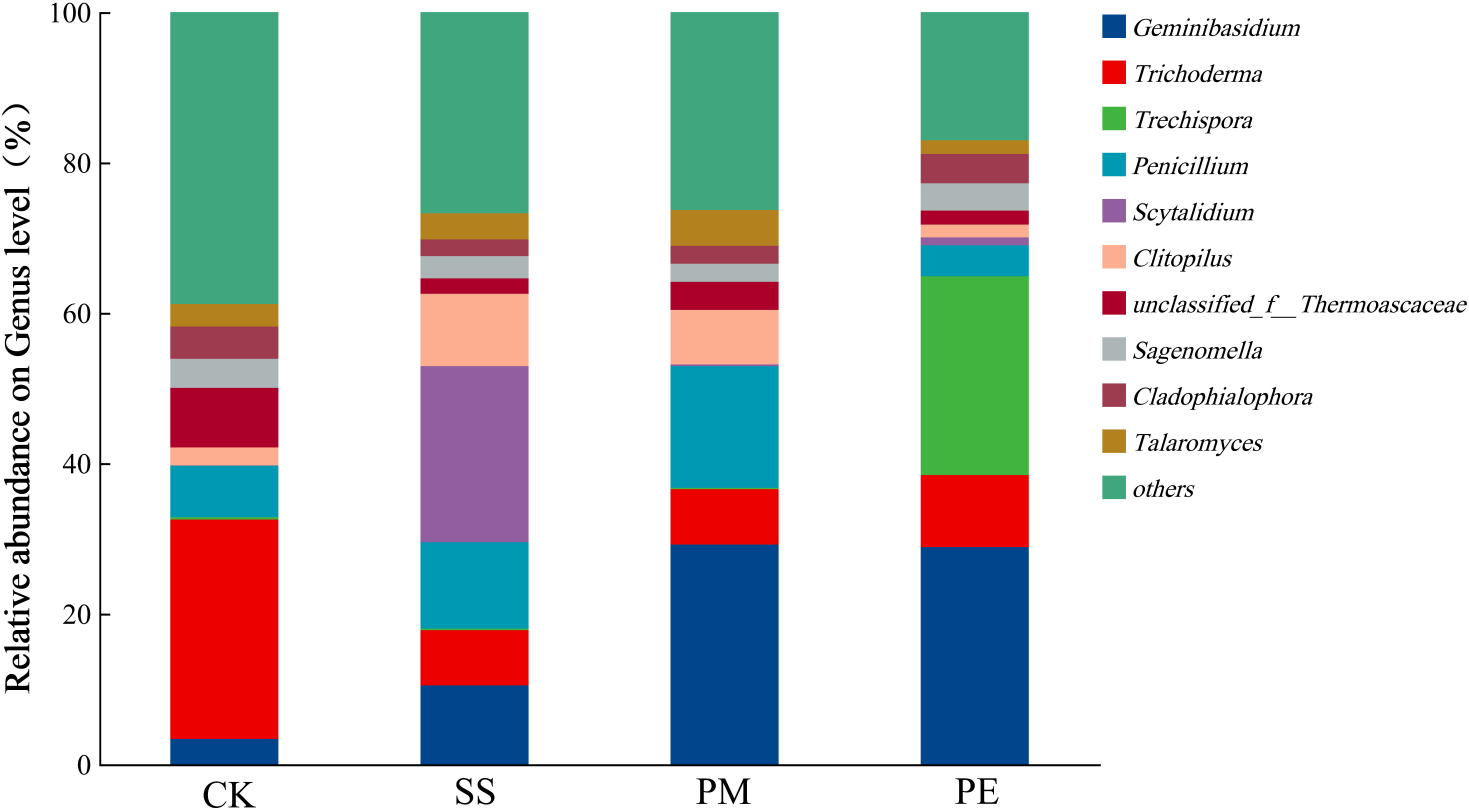
Relative abundance of soil fungi at the genus level under different fallen log types. Ck, Control (soil group without deadwood); SS, *Schima superba* (soil group beneath *Schima superba* deadwood); PM, *Pinus massoniana* (soil group under *Pinus massoniana* deadwood); PE, *Phyllostachys edulis* (soil group beneath *Phyllostachys edulis* deadwood).

At the genus level, the dominant fungal genera were *Geminibasidium*, *Trichoderma*, *Trechispora*, *Penicillium* and *Scytalidium.* After 6 years of decomposition, the relative abundances of *Geminibasidium*, *Trechispora*, *Penicillium*, and *Scytalidium* increased, while that of *Trichoderma* decreased. *Geminibasidium* had the highest relative abundance beneath PM deadwood (29.19%), closely followed by PE (28.85%) and SS (10.50%). *Trechispora* reached peak abundance beneath PE deadwood, and *Scytalidium* was more abundant beneath SS deadwood (Figure 6 and Supplementary Table 5).

### 3.3 Trophic modes and functional groups

**FIGURE 7:**
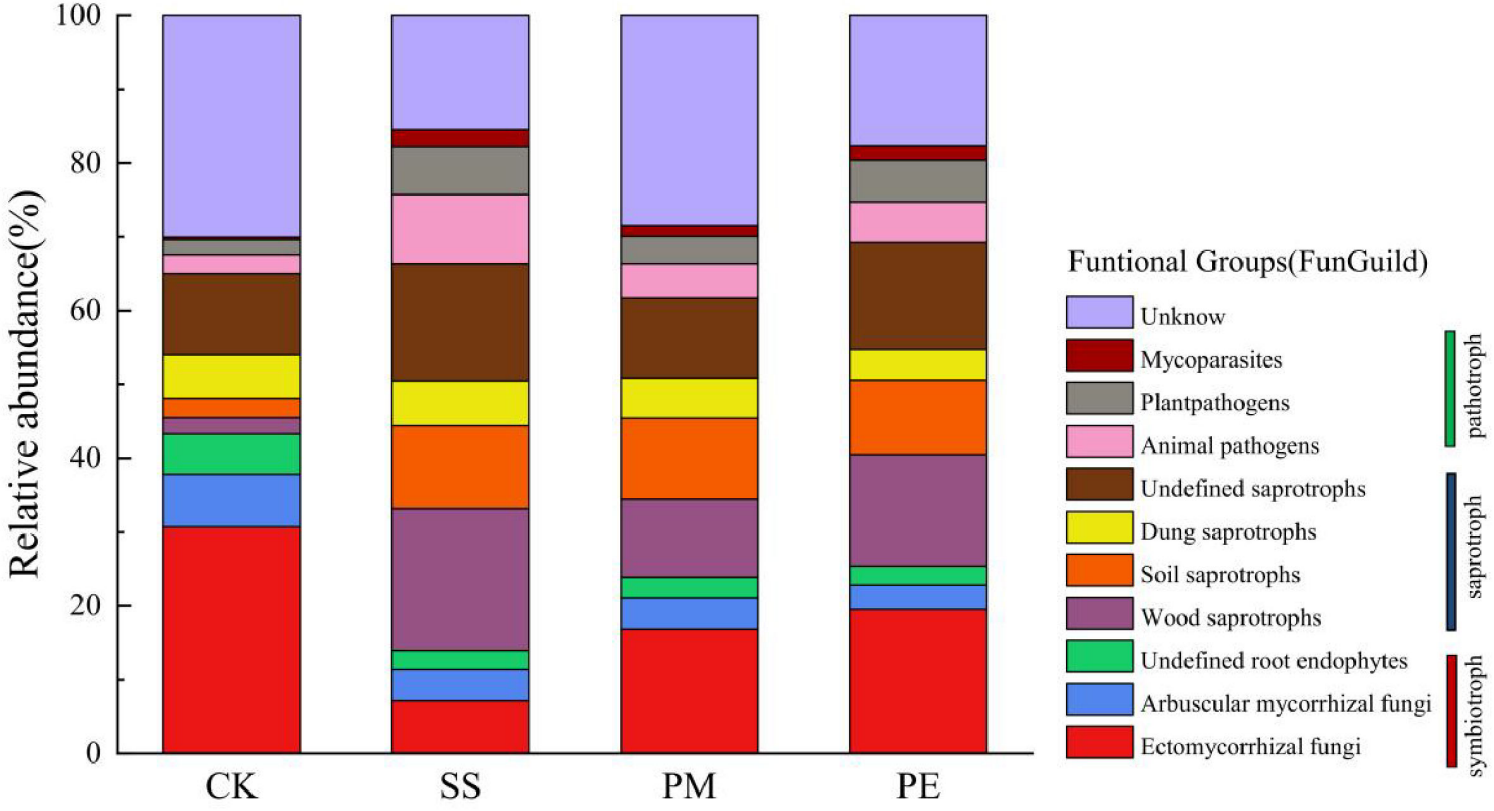
Fungal functional groups inferred by FUNGuild: variation in the relative abundance of pathotroph, saprotroph, and symbiotroph under different fallen log types. Ck, Control (soil group without deadwood); SS, *Schima superba* (soil group beneath *Schima superba* deadwood); PM, *Pinus massoniana* (soil group under *Pinus massoniana* deadwood); PE, *Phyllostachys edulis* (soil group beneath *Phyllostachys edulis* deadwood).

Fungal ASVs were classified into distinct trophic groups, which were further subdivided into specific ecological guilds. This classification relied on manually curated designations from FunGuild, a recently developed fungal classification tool that provides rigorously defined, well-documented trophic groups assignments (Nguyen et al., 2016). The ecological guilds identified in this study are shown in Figure 7. Soil fungal communities associated with logs of different tree species included three functional nutritional types: saprotrophic, symbiotic and pathogenic. Among these, symbiotic (13.98%–43.35%) and saprotrophic (21.66%–52.38%) types were predominant. After 6 years of decomposition, symbiotroph relative abundance decreased in soil while saprotroph abundance increased. Ten major fungal functional groups were detected across symbiotroph, saprotroph, and pathotroph trophic groups (Supplementary Table 6). CWD decomposition significantly decreased the relative abundances of ectomycorrhizal fungi and arbuscular mycorrhizal fungi, while increasing those of soil saprotrophs and wood saprotrophs.

### 3.4 Relationship between soil properties and fungal communities

The fungal Chao1 richness index was significantly positively correlated with MBN (*r* = −0.652, *P* < 0.05) and negatively correlated with pH (*r* = −0.652, *P* < 0.05). The Shannon index showed a highly significant negative correlation with soil pH (*r* = −0.860, *P* < 0.01) and a significant positive correlation with MBC (*r* = 0.675, *P* < 0.05), along with a highly significant positive correlation with MBN (*r* = 0.946, *P* < 0.01). In contrast, the Simpson index was significant positively correlated with soil pH (*r* = 0.694, *P* < 0.05) and highly significant negatively correlated with MBN (*r* = −0.890, *P* < 0.01) and MBC (*r* = −0.676, *P* < 0.05) (Supplementary Table 7).

**FIGURE 8:**
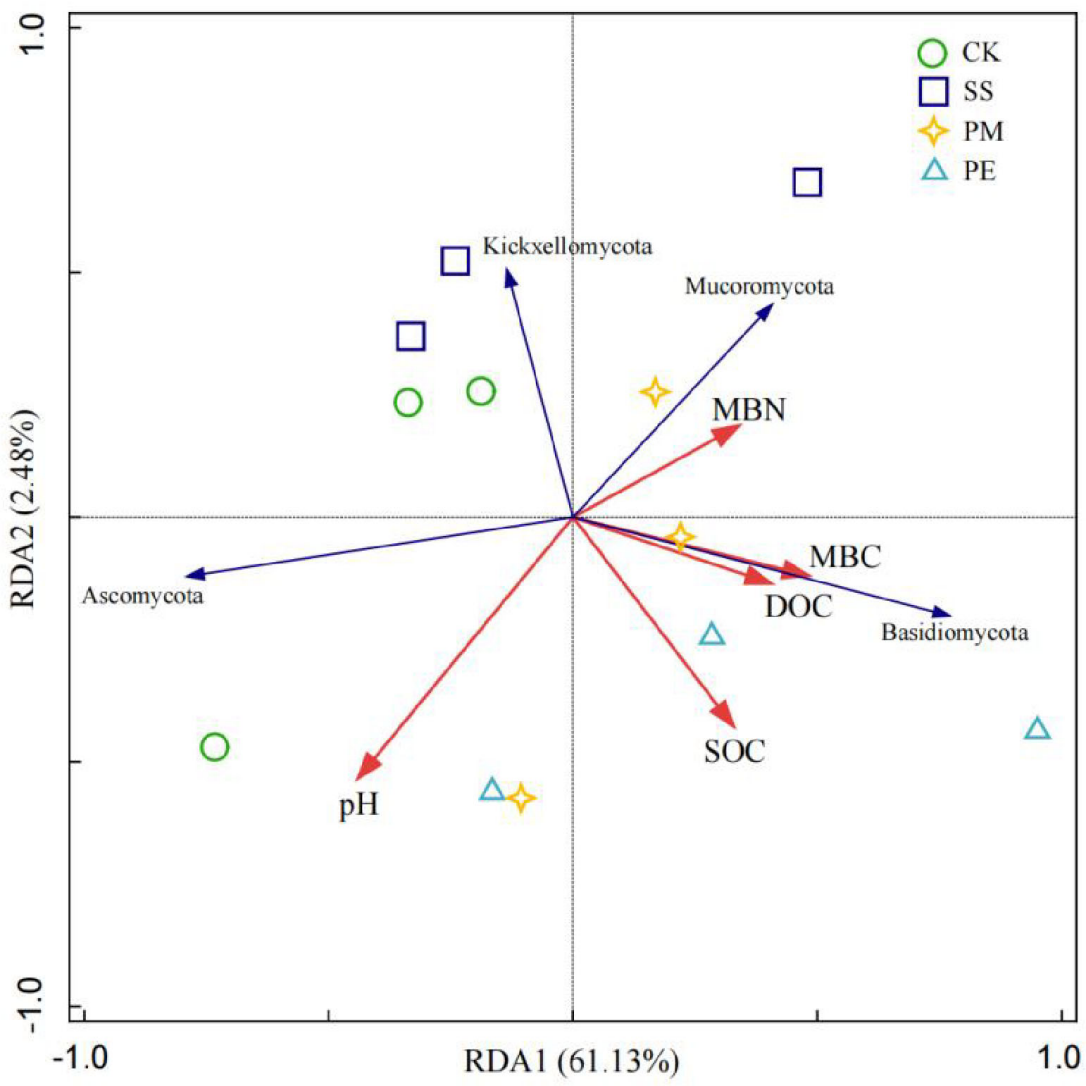
Redundancy analysis (RDA) of soil fungal phyla and soil environmental factors. Ck, Control (soil group without deadwood); SS, Schima superba (soil group beneath Schima superba deadwood); PM, Pinus massoniana (soil group under Pinus massoniana deadwood); PE, Phyllostachys edulis (soil group beneath Phyllostachys edulis deadwood).

**FIGURE 9:**
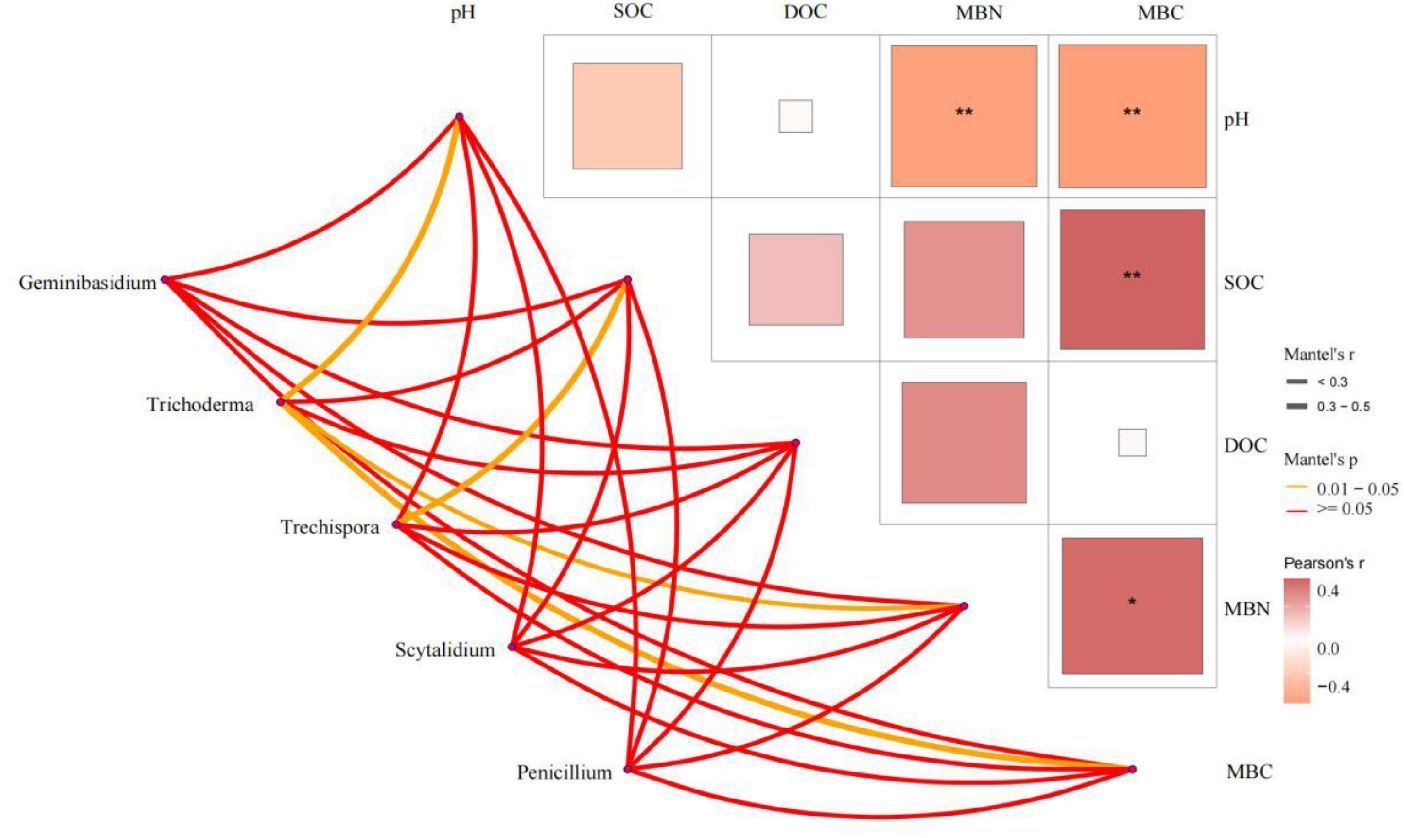
Mantel test analysis of soil fungal genus and soil properties. Ck, Control (soil group without deadwood); SS, Schima superba (soil group beneath Schima superba deadwood); PM, Pinus massoniana (soil group under Pinus massoniana deadwood); PE, Phyllostachys edulis (soil group beneath Phyllostachys edulis deadwood).

Soil variables explained 63.6% of the total changes in soil fungal communities at the phylum level (Figure 8). Monte Carlo permutation tests (*P* < 0.05) showed that soil fungal communities at the phylum level were strongly related to SOC and pH. At the genus level, Mantel test results (Figure 9) showed soil pH, MBN and MBC had significant positive correlations with *Trichoderma* (*P* < 0.05), while SOC had a significant positive correlation with *Trechispora* (*P* < 0.05) (Supplementary Table 8).

## 4 Discussion

### 4.1 Impact of decomposition on soil carbon

Over the 6-year decomposition period, we observed an overall increase in DOC and SOC content, with a significant increase beneath moso bamboo CWD. Although DOC accounts for only 0.04%–0.22% of SOC, it is the most active fraction and readily utilized by soil microorganisms. Adsorption of DOC by soil particles is a primary mechanism for sequestering organic carbon in deeper soil layers. CWD is a crucial source of DOC input to soil, and DOC concentrations are generally higher in forest stands with deadwood than in those without (Hollands et al., 2022). This DOC increase contributes to soil carbon sequestration, a phenomenon supported by other research (Wiebe et al., 2014) and corroborated by our findings.

Previous studies have shown that approximately 35% of deadwood mass is ultimately converted into microbial biomass, predominantly fungal biomass (e.g., Basidiomycetes) (Khan et al., 2016). Soil organic carbon is significantly influenced by microbial anabolic metabolism, and the stable fraction of SOC may be primarily derived from microbial biomass. Through iterative processes of cell generation, population growth, death, and decay, soil microorganisms produce large amounts of microbial residue carbon. This residue carbon remains stable in soil, enhancing contribution to the soil organic carbon pool (Yuan et al., 2021). Recent studies have quantified that microbial residue carbon can made up more than half of soil organic carbon in global forest ecosystems (Shao et al., 2019). Thus, decomposing deadwood increases soil microbial biomass (Table 1), leading to greater carbon sequestration in forest soils.

The significant SOC increase beneath moso bamboo CWD indicates its decomposition contributes more effectively to soil carbon sequestration than that of other species. This enhanced sequestration is due to the unique properties of moso bamboo, such as low lignin content and high nitrogen levels, which facilitate more efficient microbial decomposition. The resulting high-quality litter, which is characterized by a low carbon-to-nitrogen (C/N) ratio, promotes greater microbial biomass growth and increases the transformation of organic carbon into stable SOC forms (Kooch et al., 2023).

### 4.2 Influence of decomposition on fungal community diversity and composition

The slow decomposition of CWD played a crucial role in maintaining continuous organic matter supply to soil, which in turn affected soil fungi community structure. After 6 years of decomposition, fungal community α-diversity increased in soils beneath CWD (Figure 3b, c). Most fungi are symbiotic or saprophytic, so the availability of nutrient substrates is a primary determinant of fungal abundance (Prescott and Grayston, 2013). Yao et al. (2017) reported that abundant soil carbon and nitrogen sources facilitated soil fungal propagation, increasing populations of certain fungal species and promoting the coexistence of diverse fungal communities. Therefore, increased MBC and MBN after decomposition may explain the higher fungal richness and diversity observed. β-diversity analysis showed significant differences in community composition between soils with and without CWD (Figure 4). Consistent with previous research, CWD decomposition exerts a synergistic effect on fungal community structure, mediated by changes in litter input and soil properties, such as pH, C, and N content (Prescott and Grayston, 2013).

Although fungal community composition in soils beneath CWD of different tree species was similar at phylum and genus levels, their relative abundances differed significantly. Ascomycota and Basidiomycota were the dominant phyla, with *Geminibasidium*, *Trichoderma*, *Trechispora*, and *Penicillium* as the dominant genera at the study sites. These results indicate that Ascomycota and Basidiomycota were common in subtropical forest fungal communities, which aligns with previous findings (Li et al., 2022). Moreover, Ascomycota and Basidiomycota act as key soil decomposers, participating in the decomposion of recalcitrant lignified plant materials and contributing to nutrient cycling (Lauber et al., 2008; Riley et al., 2014).

Due to their specific functional traits and survival strategies, dominant fungi responded differently to CWD decomposition (Figures 5, 6). CWD decomposition was associated with observable differences in taxonomic composition, specifically an increase in the Basidiomycota-to-Ascomycota ratio. Early decomposition stages favor r-strategists such as the Ascomycota genus *Trichoderma*, recognized by traits like fast colonization, high reproductive rate and adaptation to resource-rich environments, due to high resource availability supporting their rapid growth (Bastian et al., 2009; Ali et al., 2018). After 6 years of decomposition, soil organic carbon content increased, altering the types of carbon substrates available to microorganisms. Accumulation of recalcitrant compounds (e.g., lignin) reduced the competitive advantage of some Ascomycota taxa, leading to decreased relative abundance, likely due to their lower efficiency in degrading complex lignin-rich materials. In contrast, Basidiomycota become more prevalent due to their ability to degrade complex lignin-rich materials (Li et al., 2018). Notably, accumulated recalcitrant carbon (e.g., lignin) was associated with a fungal community composition that favored taxa with K-strategy traits, such as the Basidiomycota genus *Trechispora*, which produces lignin-degrading enzymes (laccases, peroxidases), enabling them to utilize recalcitrant carbon substrates (Ferreira de Araujo et al., 2017). However, that not all microbial taxa fit neatly into discrete r- or K-strategist categories. Microbial life-history strategies often exist along a continuous gradient, yet using ecological strategy-based classification remains a valuable framework for interpreting microbial community dynamics (Peng et al., 2022).

Fungal genera distribution reflects their ecological preferences and functional roles in CWD decomposition. Previous studies have shown that *Geminibasidium*, a saprophytic fungus, thrives in high-fertility ecosystems (Pulido-Chavez et al., 2021). It can degrade cellulose, hemicellulose, and lignin, playing an important role in forest ecosystems. CWD decomposition produces leachates and fragmented materials that infiltrate soil, providing a favorable growth environment and promoting *Geminibasidium* proliferation. After 6 years of decomposition, *Geminibasidium* abundance beneath PE deadwood was significantly higher than SS (Supplementary Table 5). Moso bamboo secretes specific chemicals that suppress competing fungal species, indirectly helping *Geminibasidium* establish dominance. Furthermore, *Geminibasidium* can degrade specialized organic compounds in bamboo wood tissue, such as bamboo lignin and celluloset (Xue et al., 2023). This capability gives it an ecological niche advantage during PE decomposition, promoting its sustained growth and reproduction.

*Trichoderma* is a well-known cellulolytic decomposer, proficient at secreting hydrolytic enzymes (cellulases, hemicellulases) and oxidative enzymes (laccases and peroxidases). These enzymes enable the breakdown of cellulose and lignin in decaying wood (Othman et al., 2021). Beyond enzymatic activity, *Trichoderma* has strong antagonistic properties, competing with soil-borne pathogens, by secreting bioactive secondary metabolites. These metabolites include antibiotics, toxins, and volatile organic compounds that suppress the growth of pathogenic fungi, bacteria and other microorganisms, helping *Trichoderma* secure a competitive ecological niche in decaying wood (Dutta et al., 2023). This competitive ability is key to its role as a dominant wood-decomposing microorganisms. Furthermore, *Trichoderma* can form symbiotic relationships with other microbes, enhancing the overall microbial community involved in wood decay. It facilitates the availability of simple organic compounds and essential nutrients through primary decomposition, promoting the growth of other microorganisms and strengthening the microbial network for woody biomass breakdown (Cao et al., 2022).

In contrast, *Trechispora*, a genus of Basidiomycota, is a white-rot fungus that primarily secretes lignin-degrading enzymes (laccases and peroxidases) (Dashora et al., 2023). These enzymes effectively decompose lignin, a complex aromatic polymer in wood, contributing to efficient woody breakdown (Kumar and Chandra, 2020). *Trechispora* activity plays a crucial ecological role in decayed wood degradation and forest nutrient cycling. Increased cellulases activity and lignin availability favored the propagation of *Trechispora* (Lima et al., 2024). Therefore, *Trechispora* (Basidiomycota, K-strategist) relative abundance increased significantly beneath PM deadwood (Figure 6). Conversely, *Trichoderma* (Ascomycota, r-strategist) relative abundance decreased during decomposition, indicating these fungi are affected by ecological niche competition. This trend suggests successional replacement by more functionally efficient, better-adapted decomposers (Fukasawa and Matsukura, 2021).

Increased of *Penicillium* abundance after CWD decomposition is due to its physiological adaptability and ecological strategies to exploit altered conditions in decomposing wood. *Penicillium* can metabolize a wide range of simple organic compounds from complex lignocellulosic breakdown. It produces diverse degradative enzymes, including cellulases, hemicellulases, and pectinases, that facilitate further decomposition of residual organic matter. Furthermore, its capacity to outcompete other microorganisms through rapid growth and antimicrobial secondary metabolites makes it an essential secondary decomposers in forest ecosystems. Similarly, increased *Scytalidium* abundance is attributable to its specialized enzymatic capabilities for efficient degradation of complex lignocellulosic materials. Additionally, its competitive interactions (e.g., producing antimicrobial compounds to inhibit other microorganisms) and effective dispersal (prolific spore production and spread) support its successful colonization and proliferation in these ecological niches.

At the genus level, Mantel tests further confirmed edaphic control over key decomposers. Soil pH, MBN and MBC were all positively correlated with *Trichoderma* relative abundance (*P* < 0.05), while SOC showed a significant positive association with *Trechispora* (*P* < 0.05; Supplementary Table 8). These relationships suggest that *Trichoderma* thrives in conditions with relatively high microbial biomass and moderately acidic pH, where abundant C and N support its fast-growing, r-selected life strategy. In contrast, the significant affinity of *Trechispora* for SOC-rich microsites reflects its K-selected, lignin-degrading ability to use more recalcitrant substrates from late stage deadwood decomposition (Liu et al., 2022). Thus, the heterogeneous distribution of soil properties beneath decomposing logs acts as an environmental filter, shaping niche differentiation among dominant fungal genera and reinforcing successional turnover from opportunistic to specialized decomposers.

### 4.3 Influence of decomposition on fungal trophic modes and functional groups

As inferred from FUNGuild, our results indicate a difference in fungal community composition, with a higher relative abundance of taxa putatively associated with saprotrophic lifestyles, and suggest that these FUNGuild-inferred saprotrophic functional groups may play a key role in decomposition. Fungi form phylogenetically and functionally diverse communities with distinct trophic modes and functional groups (Schmidt et al., 2019). In this study, Symbiotroph and Saprotroph accounted for the highest proportion among all trophic modes (Figure 7). Saprotrophs are key mediators of nutrient cycling at the soil-litter interface. As primary decomposers of plant litter, they facilitate organic matter breakdown, which plays a critical role in carbon cycling and nutrients mobilization (van der Wal et al., 2013; Schmidt et al., 2019).

Coarse woody debris decomposition increases the abundance of saprotrophic fungi while reducing that of symbiotic fungi, suggesting a mechanism analogous to the Gadgil effect where competition for limiting nutrients plays a central role. In environments without deadwood, saprotrophic and ectomycorrhizal fungi occupy overlapping ecological niches. Both groups utilize extracellular enzymes to hydrolyze complex organic compounds, releasing nutrients that support fungal growth and metabolic activity (Nicolás et al., 2019). This overlap leads to intense competition and antagonistic interactions, often resulting in ectomycorrhizal fungi suppressing the proliferation of saprotrophic fungi (Smith and Wan, 2019). However, as CWD decomposition progresses, compounds such as lignin leach into the soil, alleviating nutrient limitations for saprotrophic fungi and stimulating their activity. Moreover, previous studies have shown increased soil organic matter positively affects saprotrophic fungi (Li et al., 2021). Therefore, the higher SOC beneath CWD likely promotes greater relative abundance of saprotrophic fungi, and this trend toward saprotrophs aligns with increased SOC availability though direct validation is needed.

### 4.4 Main drivers of the fungal community after CWD decomposition

Redundancy analysis revealed a significant correlation between differences in fungal composition and key soil properties, particularly soil pH and SOC. Soil pH plays a pivotal role in shaping fungal communities by modulating nutrient acquisition across the plasma membrane, influencing enzymatic secretion, and affecting the formation of mycorrhizal associations (Deacon, 2005). Additionally, pH levels can alter various soil chemical parameters, including ion concentrations, base cations, and phosphorus availability. For example, increase pH can reduce phosphorus bioavailability by inhibiting the production and secretion of extracellular enzymes that soil microorganisms need hydrolysis organic phosphorus compounds, which in turn affects fugal community composition and structure (Rousk et al., 2010; Carrino-Kyker et al., 2016).

After 6 years of CWD decomposition, SOC emerged as an important regulator of fungal communities (Figures 8, 9). SOC often reflects labile carbon availability, which drives soil microbial activity and is also a crucial factor for fungal community diversity (Wang et al., 2023). The SOC pool has two primary components: plant-derived carbon and microbial-derived carbon. Plant-derived carbon mainly exists as DOC, which includes active, non-structural, small molecular soluble organic compounds (Whalen et al., 2022). CWD is widely recognized as a substantial source of soil DOC inputs. Leaching fluxes from decomposition CWD increase DOC concentrations, particularly adding labile C to soil (Hafner and Groffman, 2005), and contribute to soil carbon sequestration (Wiebe et al., 2014). Consequently, increased SOC led to differences in dominant taxa and triggered reshuffling of the fungal community after decomposition (Jirout et al., 2011).

### 4.5 Limitations of functional inference

It is important to note that our interpretation of fungal functional guilds relies entirely on the FUNGuild database, which assigns ecological roles based on taxonomic identity rather than direct functional assays. These assignments are probabilistic and may not reflect the actual physiological capabilities or *in situ* activities of the detected fungal taxa. For example, many fungal species exhibit functional plasticity or remain poorly characterized, leading to potential misclassification. Furthermore, FUNGuild does not account for intraspecific functional variation or context-dependent expression of traits. Thus, our conclusions regarding changing in trophic modes (e.g., saprotrophy vs. symbiosis) should be interpreted cautiously as putative trends rather than definitive functional changes. Future studies should complement sequencing-based approaches with enzyme activity assays such as cellulase, laccase and peroxidase assays or metatranscriptomics to directly assess functional capacities.

## 5 Conclusion

We evaluated the effects of different tree species on soil fungal communities over a 6-year period after tree deposition using a controlled simulation experiment that eliminated environmental heterogeneity. Our findings showed that CWD decomposition and the specific tree species significantly influenced soil properties, fungal community abundance, life strategies, and trophic modes at the soil-decaying log interface. Decomposition increased fungal abundance and diversity, altered community structure and induced differences in fungal trophic modes. These changes suggested a trend of trophic modes shifting from symbiotrophic to saprotrophic fungi, reflecting the interplay between microbial communities and chemical properties of CWD-derived inputs. Changes in soil pH and SOC were key factors driving fungal community alteration. Furthermore, results underscore the ecological significance of tree species in shaping microbial community assembly and SOC stabilization. Notably, decomposition of moso bamboo deadwood promoted MBC accumulation and enhanced SOC stability, suggesting that tree species-specific traits and decomposition rates are critical determinants of soil carbon dynamics. The study improves understanding of soil fungal responses to CWD decomposition and clarifies their roles in forest carbon cycling and microbial assembly. It highlights the need to integrate tree species-specific traits and fungal functions into forest management to enhance carbon sequestration and soil health. Future research should address long-term CWD-microbe interactions across forest types and ecological gradients.

## Author contribution

NW: Conceptualization, Project administration, Supervision, Writing – original draft, Writing – review & editing. BD: Formal analysis, Methodology, Writing – original draft. RZ: Methodology, Writing – review & editing. HuiC: Methodology, Writing – review & editing. TX: Methodology, Writing – review &editing. SB: Writing – review & editing. HuaC: Writing – review &editing. XP: Writing – review & editing.

## Data Availability

The datasets presented in this study can be found in online repositories. The names of the repository/repositories and accession number(s) can be found in the article/Supplementary material.

## Footnotes

1
https://unite.ut.ee/
