# Supplementary material for: The six-year decomposition of coarse woody debris drives shifts in soil fungal communities in subtropical forests
Source: Front Microbiol. 2025 Sep 22;16:1544163. doi: 10.3389/fmicb.2025.1544163 (PMC12497771; doi:10.3389/fmicb.2025.1544163)
Supplement: Supplementary file 1 [file Data_Sheet_1.docx]

**Table S1.** The initial wood chemical and stoichiometric traits of different tree species. Small letters indicate statistically significant differences among tree species by ANOVA and post-hoc tests. PM: *Pinus massoniana*; PE: *Phyllostachys edulis*; SS: *Schima superba.*

| Traits | PM | PE | SS |
| --- | --- | --- | --- |
| Density (g cm-3) | 0.429(0.070)b | 0.615(0.128)a | 0.581(0.096)a |
| C (%) | 48.737(1.313)ab | 40.413(0.723)b | 43.068(0.113)ab |
| N (mg/g) | 3.068(0.066)b | 4.130(0.047)a | 1.480(0.006)c |
| P (mg/g) | 0.910(0.062)ab | 1.135(0.038)a | 0.826(0.017)ab |
| C/N | 16.026(0.543)b | 9.801(0.176)c | 29.101(0.096)a |
| N/P | 3.784(0.307)a | 3.737(0.149)a | 1.808(0.038)b |
| Hemicellulose (mg/g) | 227.861(3.98)b | 237.620(8.638)b | 324.860(2.203)a |
| Cellulose (mg/g) | 483.439(4.643)a | 497.971(5.356)a | 467.115(2.841)ab |
| Lignin (mg/g) | 265.000(5.092)a | 245.409(3.001)a | 179.493(3.366)c |
| Hem.+Cel. (mg/g) | 711.3(2.465)c | 735.591(4.798)bc | 791.975(2.041)a |
| Lignin/N | 8.906(0.199)b | 6.069(0.076)c | 12.481(0.223)a |
| Lignin /P | 33.224(2.652)a | 22.476(0.693)ab | 22.414(0.2)ab |

**Table S2.** Baseline characterization of the study site

| Plot | Slope/ (º) | Aspect | Altitude/m | Soil pH | SOC (mg∙kg^-1^) |
| --- | --- | --- | --- | --- | --- |
| Plot 1 | 45º-50º | Sunny side | 242 | 5.97±0.05a | 26.23±10.05a |
| Plot 2 | 45º-50º | Sunny side | 230 | 5.89±0.02b | 24.45±9.22a |
| Plot 3 | 45º-50º | Sunny side | 222 | 5.91±0.05b | 25.62±10.34a |

**Table S3.** One-way ANOVA of Soil properties (pH, SOC, DOC, MBN, MBC) between two tree species(or CWD type). PM: *Pinus massoniana*; PE: *Phyllostachys edulis*; SS: *Schima superba..*

| Soil properties | Comparison of Different Treatments | *F* | *P value* |
| --- | --- | --- | --- |
| pH | SS-PM | 12.825 | 0.023* |
|  | SS-PE | 12.394 | 0.024* |
|  | PM-PE | 6.158 | 0.068 |
| SOC | SS-PM | 1.433 | 0.297 |
|  | SS-PE | 9.179 | 0.039* |
|  | PM-PE | 5.556 | 0.078 |
| DOC | SS-PM | 25.559 | 0.007** |
|  | SS-PE | 0.118 | 0.749 |
|  | PM-PE | 113.323 | <0.001*** |
| MBN | SS-PM | 6.754 | 0.06 |
|  | SS-PE | 2.192 | 0.213 |
|  | PM-PE | 1.600 | 0.275 |
| MBC | SS-PM | 113.220 | <0.001*** |
|  | SS-PE | 52.486 | 0.002** |
|  | PM-PE | 244.492 | <0.001*** |

**Table S4.** Sequencing data statistics.PM: *Pinus massoniana*; PE: *Phyllostachys edulis*; SS: *Schima superba.*

| Pre-noise reduction sample | sequence count | ASV count | Post-noise reduction sample | sequence count | ASV count |
| --- | --- | --- | --- | --- | --- |
| CK | 145553 | 1057 | CK | 100306 | 813 |
| SS | 121389 | 1537 | SS | 114722 | 1273 |
| PM | 144987 | 1292 | PM | 114273 | 1078 |
| PE | 158528 | 1338 | PE | 118756 | 1032 |
| Total | 570457 | 5224 | Total | 448057 | 2941 |

**Table S5.** The relative abundance of soil fungi at the phylum and genus levels under different tree species treatments. PM: *Pinus massoniana*; PE: *Phyllostachys edulis*; SS: *Schima superba.*

| Phyla | CK | SS | PM | PE |
| --- | --- | --- | --- | --- |
| Ascomycota | 87.32% | 71.88% | 55.78% | 39.87% |
| Basidiomycota | 11.82% | 25.61% | 41.74% | 59.51% |
| others | 0.86% | 2.51% | 2.48% | 0.62% |
| Genus |  |  |  |  |
| *Geminibasidium* | 3.37% | 10.50% | 29.19% | 28.85% |
| *Trichoderma* | 29.16% | 7.33% | 7.36% | 9.59% |
| *Trechispora* | 0.31% | 0.21% | 0.17% | 26.45% |
| *Penicillium* | 6.84% | 11.48% | 16.21% | 4.12% |
| *Scytalidium* | 0.06% | 23.41% | 0.23% | 1.05% |
| *Clitopilus* | 2.39% | 9.62% | 7.25% | 1.71% |
| *unclassified_f__Thermoascaceae* | 7.92% | 2.06% | 3.73% | 1.86% |
| *Sagenomella* | 3.87% | 2.97% | 2.41% | 3.62% |
| *Cladophialophora* | 4.28% | 2.21% | 2.37% | 3.92% |
| *Talaromyces* | 3.01% | 3.48% | 4.76% | 1.81% |
| others | 38.79% | 26.73% | 26.32% | 17.02% |

**Table S6.** Relative abundance of functional groups within soil fungal communities under different treatment conditions. PM: *Pinus massoniana*; PE: *Phyllostachys edulis*; SS: *Schima superba.*

| Trophic mode | Funtional groups | CK | SS | PM | PE |
| --- | --- | --- | --- | --- | --- |
| Saprotroph |  | 21.66% | 52.38% | 37.85% | 43.84% |
|  | Wood saprotrophs | 2.12% | 19.22% | 10.58% | 15.13% |
|  | Soil saprotrophs | 2.64% | 11.26% | 10.99% | 10.05% |
|  | Dung saprotrophs | 5.90% | 5.97% | 5.39% | 4.18% |
|  | Undefined saprotrophs | 11.00% | 15.93% | 10.89% | 14.48% |
| Symbiotroph |  | 43.35% | 13.98% | 23.87% | 25.37% |
|  | Ectomycorrhizal fungi | 30.70% | 7.12% | 16.86% | 19.49% |
|  | Arbuscular mycorrhizal fungi | 7.09% | 4.27% | 4.26% | 3.28% |
|  | Undefined root endophytes | 5.56% | 2.59% | 2.75% | 2.6% |
| Pathotroph |  | 4.93% | 18.15% | 9.83% | 13.16% |
|  | Animal pathogens | 2.57% | 9.36% | 4.60% | 5.53% |
|  | Plantpathogens | 2.05% | 6.50% | 3.75% | 5.61% |
|  | Mycoparasites | 0.31% | 2.29% | 1.48% | 2.02% |
| Others |  | 30.06% | 15.49% | 28.45% | 17.63% |

**Table S7.** Correlation analysis between soil fungal community Alpha diversity index and soil environmental factors.

| Diversity index | pH | DOC | SOC | MBN | MBC |
| --- | --- | --- | --- | --- | --- |
| Chao1 | -0.552 | 0.340 | 0.339 | 0.675* | 0.396 |
| Shannon | -0.860** | 0.359 | 0.328 | 0.946** | 0.675* |
| Simpson | 0.694* | -0.500 | -0.452 | -0.890** | -0.676* |

**Table S8.** The correlation analysis between fungal communities and soil properties using Mantel test analysis.

| fungal | Soil properties | | | | | | | | | |
| --- | --- | --- | --- | --- | --- | --- | --- | --- | --- | --- |
|  | pH | | SOC | | DOC | | MBC | | MBN | |
|  | r | *p* | r | *p* | r | *p* | r | *p* | r | *p* |
| *Geminibasidium* | -0.126 | 0.880 | -0.111 | 0.844 | -0.075 | 0.716 | 0.014 | 0.363 | -0.070 | 0.699 |
| *Trichoderma* | 0.329 | 0.027 | 0.012 | 0.391 | -0.048 | 0.583 | 0.353 | 0.013 | 0.266 | 0.047 |
| *Trechispora* | -0.055 | 0.591 | 0.391 | 0.046 | -0.100 | 0.705 | 0.082 | 0.209 | 0.113 | 0.198 |
| *Scytalidium* | -0.047 | 0.584 | -0.042 | 0.0548 | -0.059 | 0.643 | -0.147 | 0.944 | 0.096 | 0.171 |
| *Penicillium* | -0.019 | 0.493 | 0.073 | 0.295 | -0.023 | 0.534 | -0.047 | 0.585 | -0.092 | 0.724 |


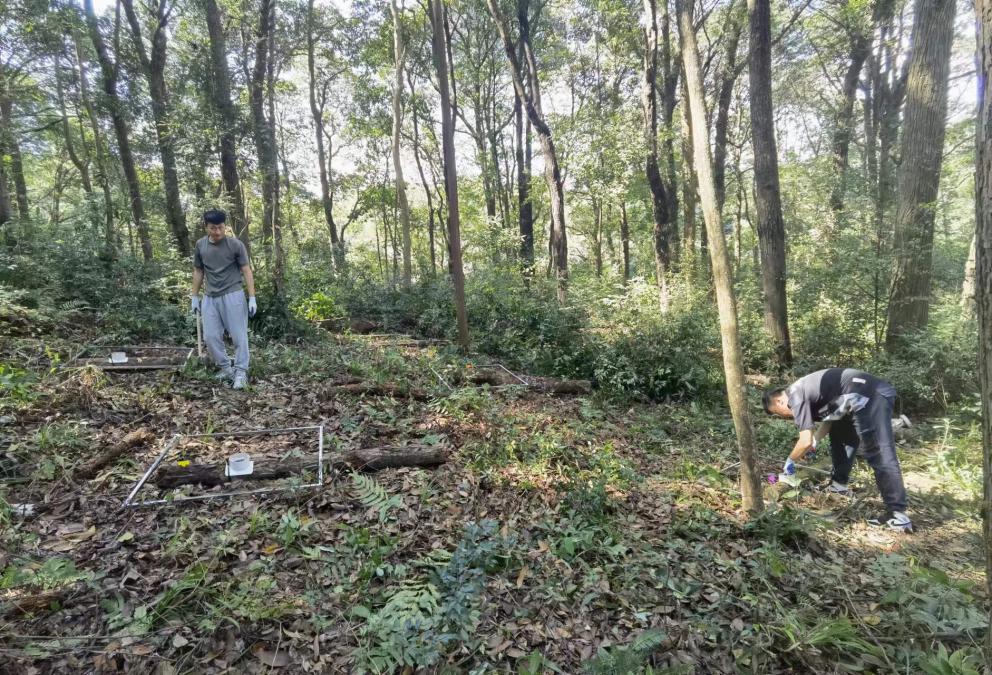


**Figure S1.** Plot establishment. Figure contains images of the author(s) only.


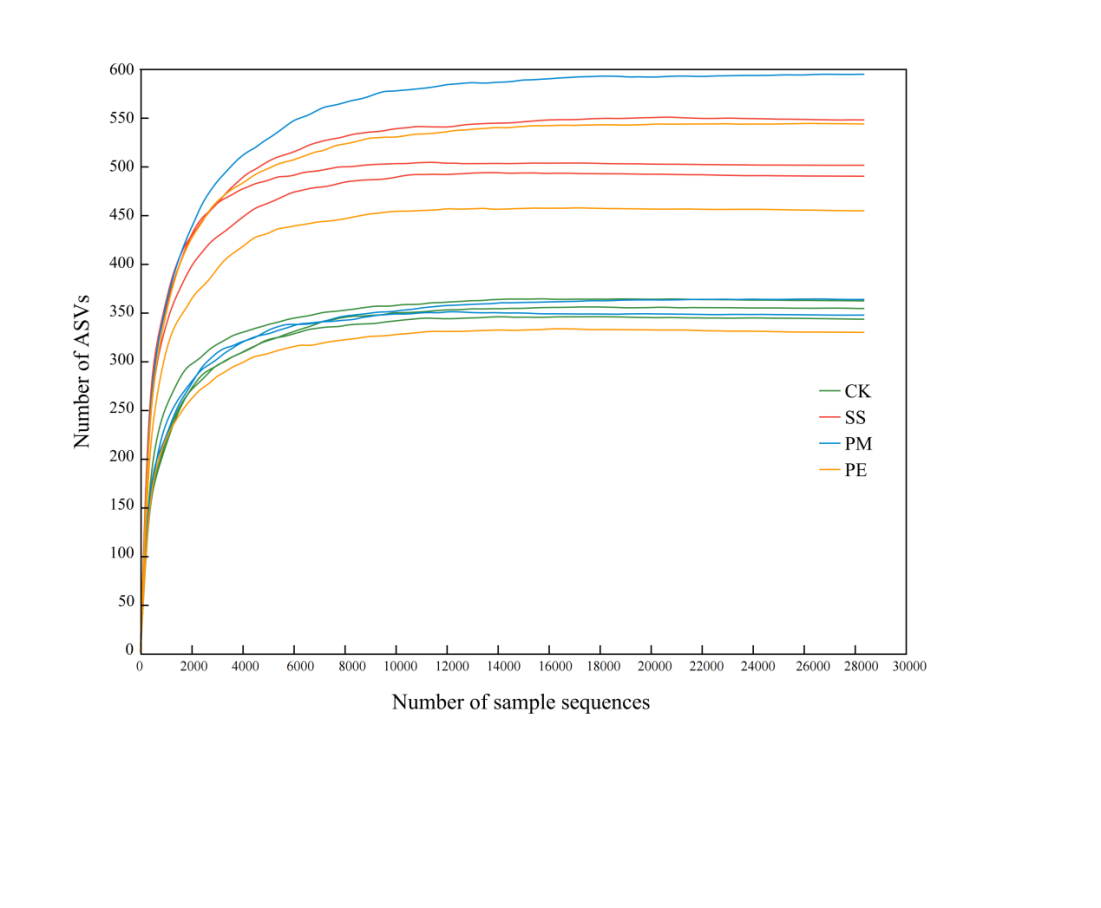


**Figure S2.** Soil fungal ASV dilution curves under the decomposition of logs from different tree species. PM: *Pinus massoniana*; PE: *Phyllostachys edulis*; SS: *Schima superba.*
